# Increase in lysophosphatidate acyltransferase activity in oilseed rape (*Brassica napus*) increases seed triacylglycerol content despite its low intrinsic flux control coefficient

**DOI:** 10.1111/nph.16100

**Published:** 2019-09-14

**Authors:** Helen K. Woodfield, Stepan Fenyk, Emma Wallington, Ruth E. Bates, Alexander Brown, Irina A. Guschina, Elizabeth‐France Marillia, David C. Taylor, David Fell, John L. Harwood, Tony Fawcett

**Affiliations:** ^1^ School of Biosciences Cardiff University Cardiff CF10 3AX UK; ^2^ Department of Biosciences Durham University Durham DH1 3LE UK; ^3^ The John Bingham Laboratory NIAB Huntingdon Road Cambridge CB3 0LE UK; ^4^ National Research Council of Canada 110 Gymnasium Place Saskatoon SK S79 0W9 Canada; ^5^ Department of Biological and Medical Sciences Oxford Brookes University Oxford OX3 0BP UK

**Keywords:** *Brassica napus*, crop improvement, flux control coefficient, lysophosphatidate acyltransferase, metabolic control analysis, storage oil, TAG, triacylglycerol

## Abstract

- Lysophosphatidate acyltransferase (LPAAT) catalyses the second step of the Kennedy pathway for triacylglycerol (TAG) synthesis. In this study we expressed *Trapaeolum majus* LPAAT in *Brassica napus* (*B. napus*) cv 12075 to evaluate the effects on lipid synthesis and estimate the flux control coefficient for LPAAT.
- We estimated the flux control coefficient of LPAAT in a whole plant context by deriving a relationship between it and overall lipid accumulation, given that this process is a exponential.
- Increasing LPAAT activity resulted in greater TAG accumulation in seeds of between 25% and 29%; altered fatty acid distributions in seed lipids (particularly those of the Kennedy pathway); and a redistribution of label from ^14^C‐glycerol between phosphoglycerides.
- Greater LPAAT activity in seeds led to an increase in TAG content despite its low intrinsic flux control coefficient on account of the exponential nature of lipid accumulation that amplifies the effect of the small flux increment achieved by increasing its activity. We have also developed a novel application of metabolic control analysis likely to have broad application as it determines the *in planta* flux control that a single component has upon accumulation of storage products.

## Introduction

Oilseed rape (*Brassica napus* L.) is one of the major oil crops, accounting currently for *c*. 15% of total global vegetable oil production (Maheshwari & Kovalchuk, 2016; Weselake *et al*., 2017). Because of the inexorable rise in demand for vegetable oils (Gunstone *et al*., 2007) coupled to limited agricultural land, efforts are continually being made to improve production. While basic characteristics of the metabolic pathway involved in oil accumulation are known (Weselake *et al*., 2009; Lu *et al*., 2011; Bates *et al*., 2013), new enzymes are still being discovered. Moreover, subtle differences in compartmentation (Chapman & Ohlrogge, 2012) and in the contribution of competing biosynthetic pathways add complexity to the overall process (Bates & Browse, 2012; Chen *et al*., 2015).

Metabolic engineering strategies can be designed using knowledge of the distribution of control within a pathway, and both theoretical and experimental insights into this can be obtained within the framework of Metabolic Control Analysis (MCA). This approach assigns a numerical value, the flux control coefficient, to the influence that a single enzyme, or a block of enzymes, can have on the metabolic flux. The value of the flux control coefficient can be used to estimate the response of a metabolic flux to overexpression of a particular enzyme (Small & Kacser, 1993; Fell, 2005). There are two major approaches in MCA: top‐down and bottom‐up. In the former, a high‐level, modular view of the metabolic pathway is obtained, and this method has another advantage in that specific ways of manipulating the activity of individual enzymes are not needed. Bottom‐up control analysis builds up the picture from the response of the metabolic system to changes in the activity of individual enzymes, using specific inhibitors or selective changes in expression.

We first applied flux control analysis to oil crops when we used top‐down control analysis to reveal that the fatty acid synthesis block of reactions is more important than that of lipid assembly in controlling overall oil accumulation in oil palm and olive (Ramli *et al*., 2002, 2005, 2009). This was also true of soybean (Guschina *et al*., 2014). Unexpectedly, we found that the situation in developing embryos from *B. napus* cv Westar was different, with some 70% of the total control lying in the lipid assembly block in this plant (Weselake *et al*., 2008; Tang *et al*., 2012). Therefore, we have concentrated our recent studies on the enzymes involved in lipid assembly to dissect further the role of different enzymes in that block. Given that our previous results have shown that control is distributed over the whole pathway, it is unlikely that overexpression of a single enzyme will, by itself, cause a substantial change in lipid flux (Fell, 2005), but measurement of the response allows estimation of the enzyme's flux control coefficient, and hence ranking of the enzymes in order of their influence.

The second reaction of the Kennedy pathway is catalysed by lysophosphatidate acyltransferase (LPAAT) (acyl‐CoA:1‐acylglycerol‐3‐phosphate acyltransferase, EC 2.3.1.51). The enzyme carries out the acylation of the *sn*‐2 position of glycerol and has two forms, one located in the plastid and the second on the endoplasmic reticulum (ER). The plastid‐localised enzyme uses acyl‐ACP as the acyl donor and has a strong preference for palmitoyl‐ACP (Frentzen, 1993). The ER isoform, conversely, is involved in triacylglycerol (TAG) production (Weselake, 2005).

The LPAAT localised to the ER has been characterised from several plants (Weselake, 2002, 2005; Wang *et al*., 2017). In contrast to the plastid‐localised enzyme, it usually has low activity with saturated acyl‐moieties. The *B. napus* LPAAT prefers oleoyl‐CoA over palmitoyl‐CoA (Berneth & Frentzen, 1990) and shows selectivity with regard to its lysophosphatidate (LPA) substrate, with those containing oleate being preferred (Oo & Huang, 1989).

In *B. napus*, LPAAT activity is the highest measured *in vitro* for the enzymes of the Kennedy pathway. Moreover, LPA accumulates at the lowest level of the three Kennedy pathway intermediates in developing seeds (Perry *et al*., 1999). This suggests that LPAAT exerts less control over carbon flux than, for example, diacylglycerol acyltransferase (DGAT) (Weselake *et al*., 2008; Taylor *et al*., 2009). Nevertheless, introduction of a mutated yeast *sn*‐2 acyltransferase gene (*SLC1‐1*), increased seed oil levels in a high erucic acid (22:1) *B. napus* variety with glasshouse growth (Zou *et al*., 1997) and in field trials (Taylor *et al*., 2002) of *c*. 9% on a per seed basis.

When two rapeseed LPAAT isoforms were expressed in the closely related Arabidopsis, they each led to a greater lipid content and seed mass (Maisonneuve *et al*., 2010). Such reports confirm a basic tenet of flux control in that all enzymes in a pathway contribute to overall regulation. The extent of this control clearly depends on the value of individual control coefficients and will also be subject to changes during various developmental stages or environmental conditions.

The work reported here shows that overexpression of a nasturtium (*Tropaeolum majus*) LPAAT in *B. napus* DH12075, which is a canola‐type low erucic acid variety, causes an increase in oil accumulation on a per seed basis, demonstrating that even an enzyme with a low flux control coefficient can make a significant impact on seed lipid biosynthesis due to the compounding effect of the exponential nature of seed TAG deposition.

## Materials and Methods

### Seeds and growth

Oilseed rape (*Brassica napus* L. var. DH12075) seed was obtained from Agriculture and Agri‐Food Canada, Saskatoon, Saskatchewan, Canada. DH12075 plants were grown at 20°C day, 15°C night with a 16 h day length in Levington M2 compost with 5 g l^−1^ slow‐release fertiliser (Levington, Surry, UK).

### LPAAT construct

Information of the original cloning and characterisation of the *Tropaeolum majus LPAAT* gene can be found in Taylor *et al*. (2010) and details of the T‐DNA structure is shown in Supporting Information Fig. S1.

### Transformation methodology

The transformation protocol has previously been described in detail (Bates *et al*., 2017). *Agrobacterium tumefaciens* AglI pBI121/NastLPAT (Fig. S1) was used directly to transform DH12075 cotyledonary petioles. Transformed shoots were selected by growth on medium containing 15 mg l^−1^ kanamycin. Plantlets were transferred to Jiffy‐7 pellets (Jiffy Products, Kristiansand, Norway) and acclimatised to growth chamber conditions, before transfer to 12 cm pots for growth to seed harvest as above.

### Genomic DNA analysis and selection of lines

DNA from transgenic lines was extracted using the method of Bates *et al*. (2017). Plantlets were verified as transgenic by PCR presence of the gene of interest and absence of the *aphIII* gene, present on the vector backbone. T‐DNA copy number estimation was determined by comparison of the *nptII* gene to an endogenous single copy gene (Bn10, Biogemma SAS) in a multiplex reaction, by quantitative PCR using the ΔΔCt method. Each sample was normalised to a known single copy control wheat line and two replicates per sample were used to determine final copy number. All reactions are carried out using ABsolute Blue qPCR ROX mix (Thermo Fisher Scientific Inc., Waltham, MA, USA), 10 μl reactions contained primers and probes (Fig. S2) at a final concentration of 10 μM using the standard run conditions for the ABI 7900 HT (Thermo Fisher Scientific).

For Southern blot analysis, DNA was isolated using cetyl trimethylammonium bromide (Stacey & Isaac, 1994), digested with *Eco*RI or *Hin*dIII and blotting was performed by the method of Glenn & Andreou (2013), using an *nptII* gene probe to determine the number of T‐DNA integration loci. T_0_ plants with a single locus insertion and one or two copies of the T‐DNA were self‐fertilised and T_1_ plants homozygous for the *T. majus* LPAAT gene identified by comparison with an endogenous single copy gene HMG I/Y (Weng *et al*., 2004) by quantitative copy number PCR using the ΔΔCt method. The *nptII* copy number was also determined in these samples as above, to compare with the expected Mendelian inheritance pattern. During the selection, null (azygous) T_1_ plants were identified as controls (see Fig. S2).

### Characterisation of seeds

The number and weight of T_2_ seeds produced and seed dimensions were recorded in all lines. Seeds were imaged and counted using a marvin seed analyser (GTA Sensorik, GmbH) and the average seed surface area calculated from the recorded images, using the marvin software. Seed TAG content was determined following two‐phase extraction (Smith *et al*., 1982), and separation by thin layer chromatography with a solvent of hexane/diethyl ether/acetic acid (80/20/1 v/v/v). Lipids were revealed by spraying with 0.1% of Floxin B and the region of the plate containing TAG scraped into a glass tube and washed with chloroform/methanol (2/1 v/v) containing 0.05% butylated hydroxytoluene. Following centrifugation at 1000 ***g*** for 5 min, the supernatant was dried under a stream of nitrogen. To the equivalent of 50 μl of extracted lipids 500 μl of 1N HCL/methanol was added and incubated at 80°C for 1 h, followed by the addition of 250 μl of hexane and 500 μl 1% KCl. After mixing and centrifugation, the upper phase was used in GC‐MS analysis. Fatty acid methyl esters were identified and quantified by capillary GC‐MS relative to C17:0 standard, using a Shimadzu GC‐2010 gas chromatograph coupled to a Shimadzu GC‐MS‐QP2010Plus single‐quadrupole MS system. Samples were introduced using split injection (split 1/50) into a capillary column (DB 23 – Agilent, 30 m × 0.25 mm, 0.15 μm film thickness). Using helium as carrier gas at a linear flow of 30 cm s^−1^, the initial column temperature of 160°C was held for 2 min, increasing to 200°C at 4°C/min, then 224°C at 6°C/min. Injector temperature was 250°C and detector temperature was 250°C.

### Detailed lipid analysis

Seeds were heated in isopropanol at 70°C for 30 min to inactivate any endogenous (phospho) lipases. A two‐phase extraction method, which had been shown to be efficient for plant tissues (Smith *et al*., 1982) was used and the lower (chloroform‐enriched) phase retained, washed with synthetic upper phase and dried down under nitrogen. Extracts were stored at −20°C under nitrogen until further analysis.

Nonpolar lipids were separated by thin layer chromatography (TLC) with a solvent of hexane/diethylether/acetic acid (80/20/1, v/v/v). Polar lipids were separated using two‐dimensional TLC, using chloroform/methanol/water (65/25/4, v/v/v) in the first dimension, followed by chloroform/acetone/methanol/acetic acid/water (50/20/10/10/5, by volume) in the second dimension. Lipid bands were revealed by spraying the plates with 0.2% (w/v) 8‐anilino‐4‐naphthalene sulphonic acid in anhydrous methanol and viewing under UV light (Smith *et al*., 1982). Standards used for chromatography were obtained from Gillingham, UK.

For analysis of acyl composition, individual lipids were scraped from the TLC plates and fatty acid methyl esters (FAMEs) prepared by acid‐catalysed methylation with 2.5% H_2_SO_4_ in dry methanol (70°C for 2 h). An internal standard of nervonic acid was used. The reaction was terminated by the addition of 5% NaCl and FAMEs were extracted into hexane and separated on a 30 m × 0.25 mm i.d. capillary column (Elite 225; Perkin Elmer, Normalk, CT, USA) using a Clarus 500 gas chromatograph with a FID detector (Woodfield *et al*., 2018). FAMEs were routinely identified by comparison of retention times with those of a GC‐411 standard (Nu‐Chek) with identities confirmed by GC‐MS (see Woodfield *et al*., 2018). Perkin Elmer totalchrom software was used for data acquisition and calculations.

### Measurement of LPAAT activity

Microsomal preparations from 27 d after flowering (DAF) embryos were isolated according to Berneth & Frentzen (1990). The microsomal pellet was re‐suspended in 50 mM Tris‐HCl buffer (pH 7.6), 5 mM dithiothreitol (DTT), 20% glycerol and stored at −80°C. Protein concentrations were determined using Bio‐Rad bovine serum albumin (BSA) Protein Assay Kit according the manufacturer's protocol.

LPAAT assays were performed according to Berneth & Frentzen (1990), using 1‐^14^C‐labelled oleoyl‐CoA, synthesised from 1‐^14^C‐labelled oleic acid (Perkin Elmer) and unlabelled 1‐oleoylglycerol‐3‐PA (LPA), using the method of Taylor *et al*. (1990). *c*. 0.2 μg μl^−1^ of microsomal protein in 50 mM Tris, pH 7.5, 1 mg ml^−1^ BSA, 500 μM oleoyl‐CoA, 500 μM LPA was incubated for 10 min at 30°C. Reaction products were separated by TLC, using chloroform/pyridine/formic acid (50/30/7 v/v/v) and quantified using a Typhoon 9400 Imager (GE Amersham Molecular Dynamics, Little Chalfont, UK).

### Radiolabelling of lipids in developing seeds

Seeds were selected for uniformity of appearance at 27 DAF. Dissected embryos were pooled, and incubations carried out with six replicates each containing five embryos. Radiolabelling incubations were carried out as in Tang *et al*. (2012).

For manipulation experiments with oleate, samples were preincubated in 1 mM oleic acid (Gillingham, Dorset, UK) dissolved in 1 mM tetramethylammonium hydroxide in 0.1 M sorbitol for 2 h with gentle shaking. Control samples contained no oleate. Samples were washed three times in 0.1 M potassium phosphate buffer (pH 7.0), 1 M sorbitol before being incubated with radioactivity.

For manipulation experiments with diazepam, 100 μM diazepam (Gillingham, Dorset, UK) was added to the radiolabelling incubation and was present for the whole 6 h of the incubation.

### Statistics

Statistical analysis of the characteristics of T_2_ seeds was carried out using 64‐bit R v.3.3.3 running under windows 10 and accessed via the rstudio development environment (R Core team, 2013). Linear mixed effect models were generated using *lme4* to describe the relationship between overexpression of the LPAAT gene in each line and the response variables of total number of seeds per plant, TAG content, seed mass and seed area (Bates *et al*., 2015); no interaction between the effects was included. The model was defined in the following way when analysing the effect of overexpressing an individual gene across all transformation events:

Trait≈Overexpression+(1|Transformation Event)

All of the generated models were tested using *car::Anova* (Fox & Weisberg, 2011). This function was used to carry out a Type II Wald chi‐squared test on each model in turn, identifying the probability that the fixed effect of overexpression on the mean value of the response variable was zero. *P*‐values given were produced using this method, with a significance threshold of *P *<* *0.05.

Error estimates for the flux control coefficients were made by simulating a thousand data sets in which each of the experimental variables entering the calculations was assigned a random value from a normal distribution centred on the mean experimental value with a variance given by the standard error of the measurement. The reported error ranges are the standard deviations of the simulated results.

### Flux control analysis

The flux control coefficient is approximately defined as the percentage change in a metabolic flux, *J*, induced by a 1% change in the activity of an enzyme *E*, all other enzymes and conditions remaining constant. For experimental evaluation, if a small change is made in an enzyme activity from an initial value *E*_0_ to *E*_1_, so that the flux changes from an initial value *J*_0_ to *J*_1_, then the flux control coefficient $C_{E}^{J}$ can be calculated as Fell (1997):

(Eqn 1) $$C_{E}^{J}=\frac{\log_{e}J_{1}-\log_{e}J_{0}}{\log_{e}E_{1}-\log_{e}E_{0}}=\frac{\log_{e}\left(J_{1}/J_{0}\right)}{\log_{e}\left(E_{1}/E_{0}\right)}$$

Here we have determined the ratio of the enzyme activities in controls and LPAAT overexpressers from LPAAT assays. However, the flux to TAG (*J*) had to be estimated from the lipid accumulation in seeds. Because this is not a linear process, but exponential (as illustrated in Notes S1), with the flux changing throughout the accumulation phase, it is necessary to show that using the exponential rate constants in Eqn 1 in place of *J* yields the same control coefficient. An outline of proof is given in Notes S1, but the formal account is in Fell (2018). The calculations use the final weights of TAG per seed and an estimate of the TAG content at the start of the exponential filling phase, derived from literature data (Notes S1).

As the calculation of the flux control coefficient depends on a flux measurement derived from the accumulation of TAG in the embryos on the plant during the whole course of seed development, the resulting value represents the coefficient in a system consisting of the whole (*in planta*) pathway from assimilation of CO_2_ to synthesis and storage of TAG in the seed. The value obtained is also intermediate between those of the controls and the overexpressors. For small changes in enzyme activity this may not be significant, but in the experiments reported here the activity has been doubled, so the value is likely to underestimate that of the controls and overestimate that in the overexpressors, given that an enzyme's flux control coefficient typically declines as its activity is increased (Kacser & Burns, 1973; Fell, 1997).

In order to compensate for the error in control coefficient estimation from large changes in enzyme activity, modified calculation methods have been devised and applied. Given that in many cases, the relationship between pathway flux and an altered enzyme activity is hyperbolic (Kacser & Burns, 1973; see also Fell, 1997), Torres *et al*. (1986) showed that the relationship between the flux control coefficient and the enzyme activity will also be hyperbolic with a parameter shared with the parameters of the enzyme‐flux hyperbola. Kruckeberg *et al*. (1989) used the Torres *et al*. (1986) function to derive an expression for the flux control coefficient values for two adjacent pairs of flux measurements, where enzyme activity had been changed by gene dosage, in terms of the ratios of the activities and the ratio of the fluxes. Small & Kacser (1993) extended this approach with their analysis of large change theory. This relates the expected fold change in metabolic flux, *f* (= *J*_1_ */J*_0_), as a result of an *r*‐fold overexpression (= *E1/E0)* of an enzyme with a flux control coefficient $C_{E}^{J}$ as:

(Eqn 2) $$f=\frac{1}{1-\frac{r-1}{r}C_{E}^{J}}$$

This can be rearranged to give the value of $C_{E}^{J}$ in the original state as:

(Eqn 3) $$C_{E}^{J}=\frac{f-1}{f}.\frac{r}{r-1}$$

Eqn 3 is equivalent to the adjacent pairs function of Kruckeberg *et al*. (1989). Because the equation also works for attenuation of enzyme activity, it is possible to take the overexpressed state as reference, yielding the following expression for the control coefficient in the overexpressed state (Notes S2):

(Eqn 4) $$C_{E+\Delta E}^{J}=\frac{f-1}{r-1}$$

Stitt's group used their version of Eqn 3 to determine the flux control coefficients of cytosolic and plastidic phosphoglucose isomerase on the fluxes of photosynthesis, sucrose synthesis and starch synthesis in *Clarkia xantiana* (Kruckeberg *et al*., 1989). They also determined control coefficients on starch synthesis of plastid phosphoglucomutase and ADP‐glucose pyrophosphorylase in *Arabidopsis thaliana* and branching enzyme in pea (Neuhaus & Stitt, 1990).

## Results

### Production and selection of LPAAT overexpressing lines

The construct used to produce overexpressing lines of *B. napus* was derived from a nasturtium (*Tropaeolum majus*) LPAAT gene (Taylor *et al*., 2010) whose activity was confirmed by complementation of a LPAAT deletion (*SLC1* ^−^) mutant and has been shown to utilise 16:0‐CoA and 18:1‐CoA with about equal efficiency (Taylor *et al*., 2010). The details of the construct are shown in Fig. S1.

Twenty‐two T_0_ transgenic plants were regenerated from three independent transformation experiments and confirmed as transformed by PCR presence of the LPAAT gene of interest, and PCR absence for the *aphII* gene. The T‐DNA copy number was determined by qPCR assay, based on the *nptII* gene and the number of T‐DNA integration loci were determined by Southern blotting using the kanamycin resistance gene, *nptII*, as the probe. T_0_ plants containing a single transgene insertion, and that produced T_1_ seeds, were selected for growth of the next generation. T_1_ plants homozygous for the transgene and *nptII* gene were identified by a combination of Southern blotting and copy number qPCR for both LPAAT and *nptII* genes with genomic DNA (Weng *et al*., 2004), as illustrated in Fig. S2. This procedure identified two T_0_ plants with independent single copy T‐DNA insertion loci (plants 2‐14 and 3‐6). Following self‐fertilisation, T_1_ plants homozygous for the T‐DNA insert from line 2‐14 (2A; 2B) and from line 3‐6 (3A and 3B) were identified for subsequent biochemical analysis, alongside null plants (2azA; 2azB; 3azA and 3azB) where segregation resulted in no LPAAT or *npt*II transgenes being present.

### Analysis of LPAAT enzyme activity and seed TAG content

First, the kinetic constants for LPAAT in microsomal preparations were determined from initial rate measurements of the accumulation of radiolabelled PA in the presence of a high concentration of one substrate and a range of concentrations of the second. A single parameter for *V*_*max*_ was determined using a bi‐substrate rate equation, from the Systems Biology Ontology (https://www.ebi.ac.uk/sbo/main/SBO:0000151), to fit both sets of substrate data simultaneously. The rate equation represents a generalised extension of the uni‐substrate Michaelis−Menten equation, and nonlinear regression using this equation gave a *V*_*max*_ value of 9.8 nmol min^−1^ mg^−1^ microsomal protein and *K*_*m*_ values of 103.1 μM for oleoyl‐CoA and 15.2 μM for LPA (Fig. S3).

**Figure 1:**
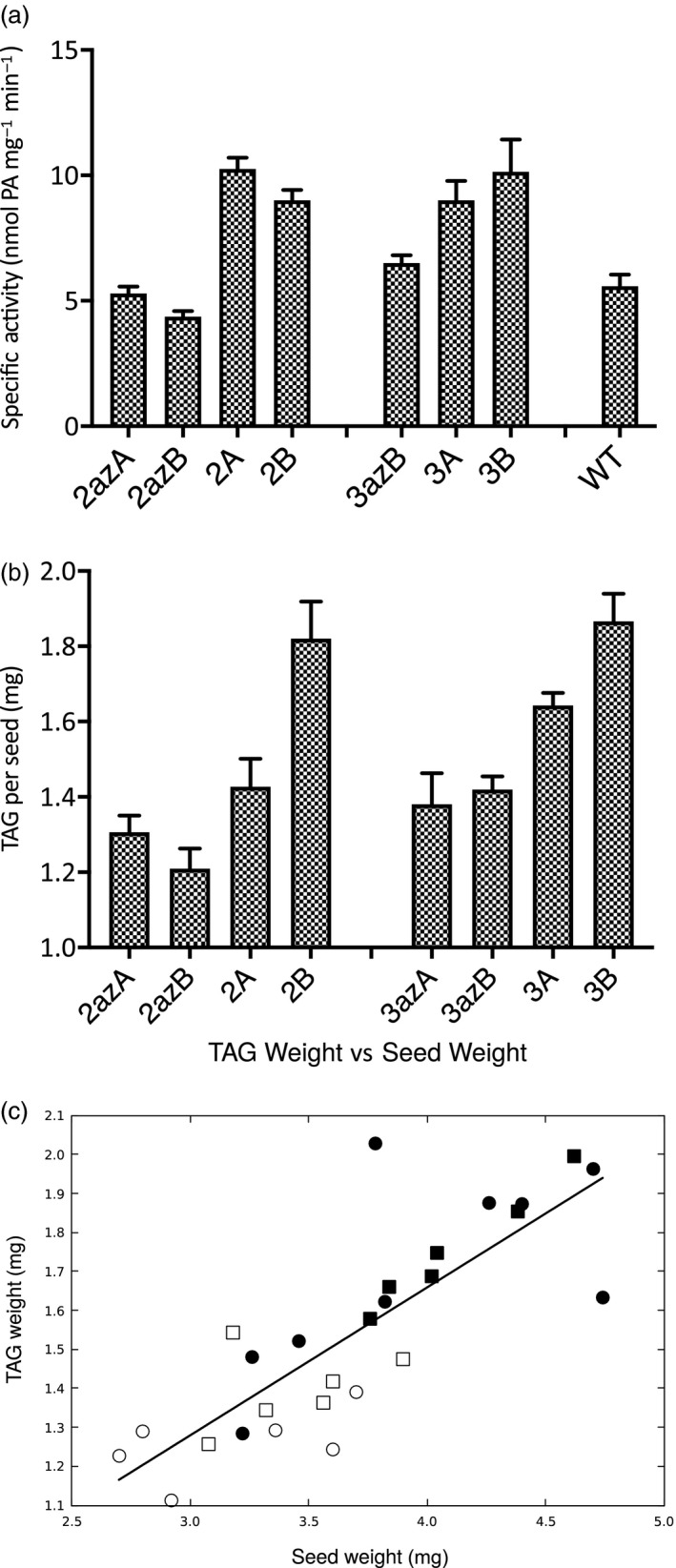
Characterisation of *Brassica napus* DH12075 seeds. (a) Lysophosphatidate acyltransferase specific activity in microsomal preparations of transgenic lines. Overexpressing lines are significantly different to their azygous controls: for plant 2 lines, *P* < 0.001 and for plant 3 lines, *P* < 0.05 by Student's *t*‐test. (b) Triacylglycerol (TAG) content per seed. Overexpressing lines are significantly different to their azygous controls: for plant 2 lines *P* < 0.01 and plant 3 lines *P* < 0.001 by Student's *t*‐test. Data shown are the mean ± SEM of three independent biological replicates. (c) Comparison of TAG per seed and seed weight: 2azA/2azB lines, open circles; 3azA/3azB lines, open squares; 2A/2B overexpressing lines, filled circles; 3A/3B overexpressing lines, filled squares. The equation of the line is *y* = 0.380*x* + 0.14, corresponding to a TAG content of 42 ± 1% over the range of seed weights, and the regression line is significant with a *P* < 0.01.

In order to evaluate the contribution of overexpression on TAG production, we measured LPAAT activity in developing T_2_ embryos (at 27 DAF, which is in the rapid phase of TAG accumulation) and determined the TAG content of mature T_2_ seeds from plants grown at the same time. There was a consistent pattern of higher activity in transgenic lines; 2A and 2B had approximately double the LPAAT activity of the wild‐type (WT) DH12075 and their azygote controls (2azA and 2azB). Lines 3A and 3B had approximately double the activity of the WT, and 30–50% greater activity than the corresponding azygote (3azB) (Fig. 1a).

The mean TAG content of mature seeds of the null controls 2azA/2azB was 1.26 ± 0.04 mg per seed and that from 3azA/3azB was 1.40 ± 0.04 mg. The average value for lines overexpressing LPAAT was 1.63 ± 0.14 mg in lines 2A/2B and 1.75 ± 0.06 mg in lines 3A/3B. These values equate to a mean increase in TAG in mature seeds of lines 2A/2B of 29% and in lines 3A/3B of 25% (Fig. 1b). Statistical analysis using linear mixed effect models showed that overexpression of LPAAT correlates to an increase in TAG content (mean effect size 0.408 mg) and seed area (mean effect size 0.573 mm^2^) in lines derived from the two different transformation events. Plotting TAG weights against seed weights (Fig. 1c) for overexpressing and azygous control plants confirms that these are highly correlated for both 2‐14 derived (*P* < 0.05), and 3‐6 derived plants (*P* < 0.01). Both azygote and overexpressing lines contain *c*. 42% TAG and the increase in TAG per seed in LPAAT overexpressors is accounted for by an increase in seed size.

### Flux responses in developing seeds

**Figure 2:**
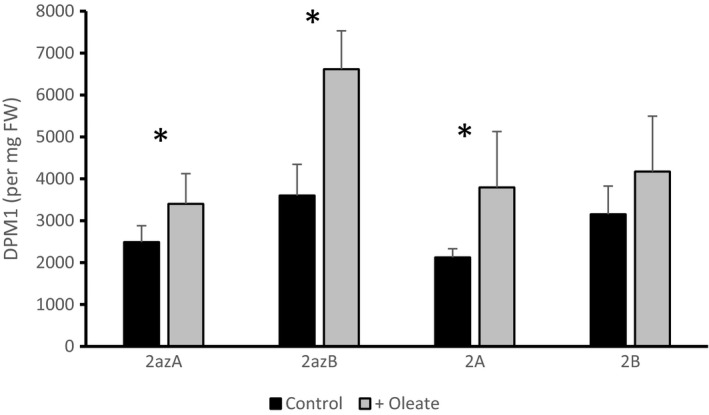
Effect of 1 mM exogenous oleate on incorporation of radioactivity from [U‐^14^C] glycerol into total lipids of 27 d after flowering (DAF) lysophosphatidate acyltransferase (LPAAT) overexpressing transgenic lines of *Brassica napus* DH12075 (2A and 2B) compared with azygote lines (2azA and 2azB). Data shown are means ± SD. (*n* = 6) with significance (Student's *t*‐test) indicated (*, *P *<* *0.05).

**Figure 3:**
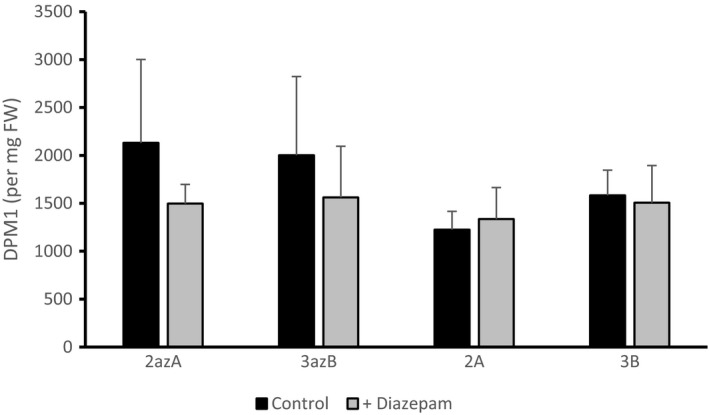
Effect of 100 μM diazepam on the incorporation of radioactivity from [U‐^14^C] glycerol into total lipids of 27 d after flowering (DAF) lysophosphatidate acyltransferase (LPAAT) overexpressing transgenic lines of *Brassica napus* DH12075 (2A and 3B) compared with azygote lines (2azA and 3azB). Data shown are means ± SD. (*n* = 6). Data were tested for significance using the Student's *t*‐test, none gave a *P*‐value < 0.05.

LPAAT catalyses the second of four reactions in the Kennedy pathway for TAG biosynthesis; this and other associated reactions can be followed using [U‐^14^C] glycerol as a precursor (Ramli *et al*.,^2002^; Guschina *et al*.,^2014^; Allen *et al*.,^2015^; Bates, 2016). In previous flux control experiments with *B. napus* L. cv Westar, we showed that exogenous oleate would increase lipid synthesis while diazepam would reduce it (see Tang *et al*., 2012 for background information on the effects of diazepam). As expected, the addition of 1 mM oleate caused relative increases in incorporation of radioactive glycerol backbone into glycerolipids, in both the azygote control plants and the two different overexpressing lines (Fig. 2). In contrast with experiments in Westar, a statistically significant difference in labelling could not be shown in either the azygote controls or the LPAAT overexpressing lines in the presence of diazepam (Fig. 3). However, a differential effect of diazepam on the distribution of fatty acids in polar lipids was seen in the presence of diazepam in azygous plants which was not evident in LPAAT overexpressors, as reported below.

### Estimation of the *in planta* flux control coefficient of LPAAT from lipid accumulation

We can make an estimate of the degree of control exerted by LPAAT over TAG accumulation because previous studies have shown that most synthesis occurs with first‐order kinetics, resulting in an exponential profile during seed development. We established common features of seed TAG accumulation from previously published data (Turnham & Northcote, 1983; Slabas *et al*., 1986, 1987; Hellyer *et al*., 1992; Fawcett *et al*., 1994). In all cases, an initial lag phase led to an exponential phase (starting when mean seed lipid content was 0.034 ± 0.0122 mg) and was followed by a period of slower TAG synthesis. In the final stages of embryogenesis – the desiccation phase ‐ TAG degradation is known to occur due to the action of SUGAR‐DEPENDENT 1 lipase (Kelly *et al*., 2013) In *B. napus* this has been measured as a *c*. 10% reduction in seed lipid content (Chia *et al*., 2005). Similar quantities of TAG appear to be synthesised and degraded following the end of exponential synthesis, resulting in a final seed TAG content close to that observed at the end of the exponential phase (Turnham & Northcote, 1983).

A partial time course of wild‐type plants used in this study confirmed that lipid accumulation was in the exponential phase for at least the period from 20 to 27 DAF, and so had the same characteristics as previous studies. Furthermore, overexpressors and azygote lines were grown together and there were no visual differences in development, as judged by the timings of flowering and seed set. Hence, as explained in Notes S1, it is possible to replace the relative fluxes to TAG by the ratio of exponential rate constants calculated from the final weights of TAG in the seeds and then apply Eqn 1 presented in the Materials and Methods to calculate the flux control coefficient of LPAAT *in planta* using the measured relative differences in LPAAT activities. The resulting values of the flux control coefficient are 0.10 for the 2–14 derived plants and 0.14 for the 3–6 derived plants (Table 1).

**Table 1:** *Brassica napus* DH12075 lysophosphatidate acyltransferase (LPAAT) flux control coefficient calculations: data and results.

Plants	Mean LPAAT activity (nmol min^−1^ mg^−1^) (*n*)	Mean TAG content (mg per seed) (*n*)	*k*∆*t*	Small change calculation $C_{E}^{J}$	Large change calculation $C_{E}^{J}$
2azB/2azA	4.83 ± 0.22 (12)	1.26 ± 0.04 (6)	3.53	0.10	0.14 ± 0.04
2A/2B	9.63 ± 0.35 (12)	1.63 ± 0.07 (6)	3.78		0.07 ± 0.02
3azB	6.51 ± 0.31 (6)	1.42 ± 0.03 (3)	3.65	0.14	0.17 ± 0.05
3A/3B	9.58 ± 0.74 (12)	1.76 ± 0.06 (6)	3.86		0.12 ± 0.04

Flux control coefficients were calculated using the small change method (Eqn 1, main paper) and the large change method (Eqns 3 and 4, main paper). The *k*∆*t* values were calculated from Supporting Information Notes S1; Eqn S1.3, using the mean lipid content at *t* = 0 of 0.034 ± 0.012 mg per embryo (*n* = 5). The errors quoted are standard errors, except for the flux control coefficients where they are the standard deviations of a simulated data set, as explained in the Materials and Methods section.

TAG, triacylglycerol.

It is likely that these values are an underestimate of the actual values in the azygote control plants because the control coefficient is calculated for a significant increase in LPAAT activity (2.0‐fold and 1.5‐fold for the 2A/2B and 3A/3B plants, respectively). On account of the generally concave relationship between flux control coefficients and enzyme activity, the result is likely to be down‐weighted by the lower value in the overexpressors.

Analysis of our flux and enzyme data confirms that it follows a hyperbolic relationship (Fig. S1.3; Notes S1) which validates the application of the large change corrections presented as Eqns 3 and 4 in the Materials and Methods section. The control coefficient for LPAAT in the azygote controls over the lipid accumulation rate is calculated as 0.14 for 2azA/2azB and 0.17 for the 3azA/3azB plants, whereas the values in the LPAAT overexpressing plants reduce to 0.07 for the 2A/2B plants and 0.12 for the 3A/3B plants, respectively (Table 1).

### Analysis of endogenous lipid classes

**Figure 4:**
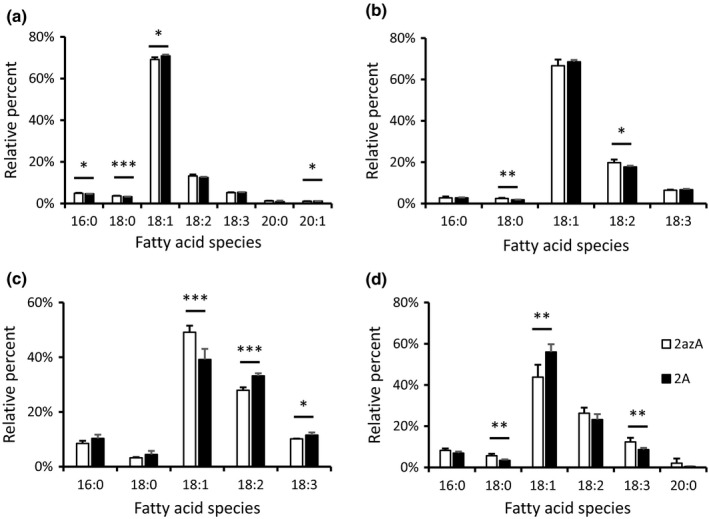
Fatty acid composition of lipid classes involved in triacylglycerol (TAG) formation in lysophosphatidate acyltransferase (LPAAT) overexpressing transgenic lines of *Brassica napus* DH12075 (2A) compared with azygote lines (2azA). Fatty acid species with over 1% content are shown for (a) TAG species, (b) diacylglycerol (DAG) species, (c) phosphatidic acid (PA) species, and (d) PC species. Data show mean ± SD (*n* = 4). (*, *P* < 0.05; **, *P* < 0.01; ***, *P* < 0.001 by Student's *t*‐test). Fatty acids are 16:0, palmitic; 18:0, stearic; 18:1, oleic; 18:2, linoleic; 18:3, α‐linolenic; 20:0, arachidic.

In order to check whether overexpressing LPAAT changed general aspects of lipid metabolism, we examined the endogenous lipids in developing seeds at 27 DAF. Data from two different overexpressing lines are compared with their equivalent azygote lines (Table 2). As would be expected for seeds midway through oil accumulation, the pattern was dominated by TAG (*c*. 90% of total lipids) followed by total polar lipids. We examined the acyl composition of a range of lipid classes where some small, but significant, differences were found. Fig. 4 shows data for triacylglycerol (TAG), diacylglycerol (DAG) and the two main phosphoglycerides directly involved in oil biosynthesis, PA and phosphatidylcholine (PC). The complete data for all individual lipids analysed is given in Table S1. Results for two independent transgenic lines compared with control azygotes were consistent and the alterations were statistically significant in at least one of the lines. For TAG, the saturated acids (palmitic and stearic) were reduced while oleate was slightly increased. The same changes were found in DAG where linolenate was also decreased. For PC, the alterations in fatty acid composition found for DAG were broadly reproduced (although the decrease in linoleate was not statistically significant). Linolenate was also reduced. In contrast to the rather consistent changes in TAG, DAG and PC, the transgenics showed decreases in the percentage of oleate and increases in linoleate and linolenate in PA. It was noticeable that the changes found in individual fatty acids in PA or PC were larger than for the nonpolar lipids, DAG and TAG. There were very few changes to the major fatty acids of the plastid lipids, mono‐ and digalactosyl diacylglycerols and phosphatidylglycerol or for phosphatidylethanolamine and phosphatidylinositol (Table S1), which are not directly involved in oil accumulation.

**Table 2:** Relative distribution of acyl lipid species of 27 d after flowering (DAF) embryos of *Brassica napus* DH12075 lines.

	3azA	3A	3azB	3B
TAG	87.3 ± 3.3	92.1 ± 1.6	92.8 ± 0.3	89.4 ± 0.6
DAG	1.6 ± 0.1	1.8 ± 0.2	1.5 ± 0.1	1.7 ± 0.1
Polar lipids	8.0 ± 0.8	4.5 ± 0.3	5.4 ± 0.2	8.6 ± 0.5
Other	3.1 ± 3.9	1.6 ± 1.8	0.3 ± 0.1	0.3 ± 0.03

Lipid species, as quantified through fatty acid amounts include triacylglycerol (TAG); diacylglycerol (DAG) and total polar lipids. *n* = 6, means ± SD. Values are expressed as a percentage of the total.

**Figure 5:**
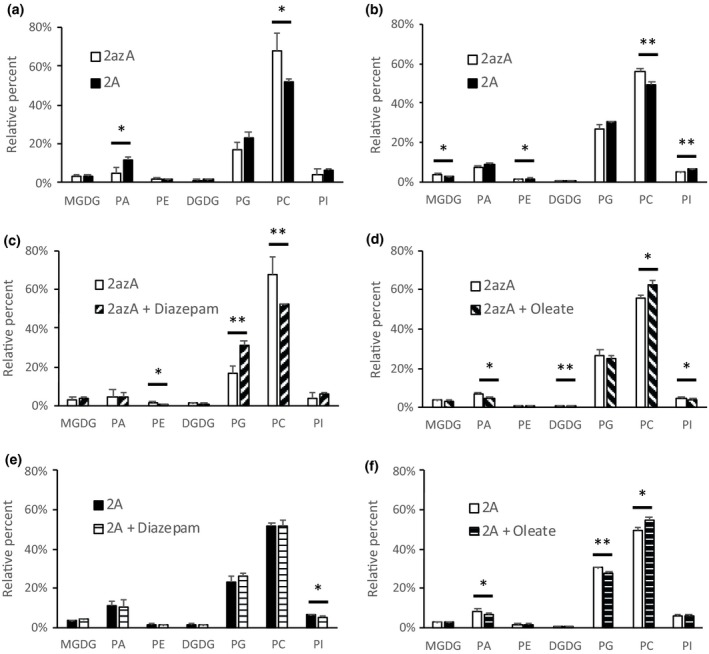
Relative distribution of ^14^C between polar lipid species following incubation of [U‐^14^C] glycerol with 27 d after flowering (DAF) embryos of lysophosphatidate acyltransferase (LPAAT) overexpressing transgenic line of *Brassica napus* DH12075 (2A) compared with azygote line (2azA) in the presence or absence of 100 μM diazepam or 1 mM oleate. (a, b) Comparison between uptake of [U‐^14^C] glycerol into LPAAT overexpressor and azygote line. (c, d) Influence of 100 μM diazepam or 1 mM oleate on an azygote line, respectively. (e, f) Influence of 100 μM diazepam or 1 mM oleate on an LPAT overexpressing line, respectively. Data show means ± SD (n = 3). (*, *P* < 0.05; **, *P* < 0.01, by Student's *t*‐test).

As a further check to examine whether LPAAT‐overexpression (OE) lines showed changes in lipid metabolism, we analysed lipid classes from our radiolabelling experiments. The data from the oleate and diazepam addition experiments (Figs 2, 3) are shown in Fig. 5. While there were no consistent alterations in the relative labelling of the nonpolar lipids (which were similar to those reported previously for cv Westar (Tang *et al*., 2012), there were changes in the polar lipids. When LPAAT‐OE lines were compared with azygote controls. PA and phosphatidylglycerol (PG) were relatively better labelled, while PC was less labelled (Fig. 5a,b).

Addition of oleate reduced the relative labelling of PA and PG but increased that of PC in both azygote controls and LPAAT‐OE lines and when diazepam was used, it reduced the relative labelling of PC and increased that of PG in azygotes (Fig. 5d). In agreement with the lack of effect of total lipid labelling in LPAAT‐OE lines (Fig. 3), the inhibitor had no effect on the relative labelling of polar lipid classes (Fig. 5c).

## Discussion

With diminishing new agricultural land and continually increasing demands for vegetable oils, efforts to increase storage oil productivity in different plants have received considerable attention, including overexpression of single Kennedy pathway enzymes (e.g. diacylglycerol acyltransferase (DGAT); Weselake *et al*., 2008; Taylor *et al*., 2009), increasing carbon supply for lipid assembly (Cernac & Benning, 2004; Liu *et al*., 2010), expanding the time period for rapid oil accumulation (Kanai *et al*., 2016) or combining a number of gene manipulations to expand the ‘push/pull’ technique (Meyer & Kinney, 2009; Vanhercke *et al*., 2013; Marillia *et al*., 2015) to include other constraining factors (e.g. Liu *et al*., 2017; Vanhercke *et al*., 2017).

Previous work with oilseed rape has shown that, while both fatty acid synthesis and lipid assembly are important for the overall control of oil accumulation, the latter reactions have a more prominent role than in other crops (Tang *et al*., 2012). While the DGAT reaction was shown to be important (Weselake *et al*., 2008), the theory of metabolic control dictates that control is distributed throughout the system (Kacser & Burns, 1973; Fell, 1997) and so we have initiated a series of investigations to examine the effect of overexpression of different enzymes involved in lipid assembly; here we determine the control residing in LPAAT.

Although LPAAT has been shown to be a high activity enzyme in the Kennedy pathway in oilseed rape embryos ensuring low levels of its substrate, lysophosphatidate, during development (Perry *et al*., 1999), some overexpression studies in various seeds have shown increased TAG accumulation (Zou *et al*., 1997; Taylor *et al*., 2002; Maisonneuve *et al*., 2010; Wang *et al*., 2017). Our studies, here, showed 25–29% increases in TAG per seed compared with their matched azygote controls (Fig. 1b), resulting from *c*. two‐fold increases in LPAAT activity (Fig. 1a). This increase in TAG exceeds the 14% increase observed from four‐fold overexpression of DGAT (Weselake *et al*., 2008), indicating that LPAAT has at least an equivalent influence on flux as DGAT.

In previous experiments with *B. napus* cv Westar, we had shown that flux control studies could detect a significant shift in control following overexpression of a DGAT1 gene (Weselake *et al*., 2008) and in a top‐down study of lipid assembly in *B. napus* Westar we were able to use both single and double manipulation experiments and both [^14^C] acetate and [^14^C] glycerol as radiolabelled precursors (Tang *et al*., 2012). In the current study, we chose to use the *B. napus* double haploid cultivar DH12075 due to ease of transformation and because Westar is not a favoured agricultural variety, due to its susceptibility to blackleg. Unfortunately, variability in radiolabelling from [1‐^14^C] acetate with cv DH12075, and the fact that labelling from [U‐^14^C] glycerol in overexpressing lines was no different to that in azygote controls, meant that it was not possible to determine accurate flux control values from *in vitro* measurements using the techniques previously employed (Tang *et al*., 2012).

Instead, we developed a novel approach to estimate the flux change in TAG synthesis caused by increasing LPAAT activity from the final weight of lipid in the seed and establishing that the kinetics of TAG accumulation are exponential. For future application of this method, it could be improved by measuring the time course of TAG accumulation in control and experimental plants in order to give a direct comparison of the exponential rate constants. It should be noted that the control coefficient obtained is measured *in planta* (from CO_2_ through assimilation, transfer of assimilate to the embryo, lipid synthesis and assembly), whereas previous *in vitro* measurements (e.g. Weselake *et al*., 2008; Tang *et al*., 2012) are confined to lipid synthesis (from acetate) and lipid assembly in isolated embryos.

Changing enzyme activity, as here, by change in gene dosage necessarily results in large relative changes and hence to a loss of accuracy in the estimate of the flux control coefficient. Therefore, we calculated a correction for this using large change theory as previously applied in plant metabolism research (Kruckeberg *et al*., 1989; Neuhaus & Stitt, 1990). However, this implicitly relies on a near hyperbolic relationship between enzyme activity and flux, which we have shown to be approximately true for our data (Fig. S1.3; Notes S1).

If relationships between the degree of overexpression and TAG accumulation continue to be hyperbolic, we can estimate that whereas further overexpression to *c*. 4 times the wild‐type activity would be expected to increase the flux to 1.1 times that in the wild‐type – a diminishing return compared with the 1.06 obtained here for a roughly two‐fold activity change – the final seed weight could increase to 1.5 times that of wild‐type. After four‐fold increase in activity, the scope for further gain in seed weight from LPAAT manipulation diminishes, but the calculation suggests that our experiments have not reached the limit for improvement. (Details given in Notes S1; Fig. S1.4.)

The summation theorem of Kacser & Burns (1973) dictates that the sum of all flux control coefficients on lipid accumulation is 1.0, and as 0.14–0.17 is contributed by LPAAT, other processes in the whole pathway together possess control amounting to 0.83–0.87, so that the value for LPAAT might appear relatively small. However, as this is an *in planta* control coefficient, the other processes include photosynthesis and generation of assimilate as well as lipid synthesis and assembly in the embryo. Previous experiments on the distribution of flux control have been performed on isolated embryos (Weselake *et al*., 2008; Tang *et al*., 2012), and so investigated the partitioning of control between fatty acyl‐CoA synthesis, from acetate (0.3), and assembly into triacylglycerol (0.7). The control exerted by these processes would be proportionately lower in a whole plant context because of the control exerted by photosynthesis and assimilation (essentially, the *source strength*). Although there are no precise determinations of the source vs sink strengths for oilseed rape, such results as there are suggest the source contribution is at least comparable to the sink strength (Hua *et al*., 2012; Wang *et al*., 2016). If source and sink were approximately equal, and assuming that the *in vitro* conditions are sufficiently similar to those of embryos *in vivo*, the control of the lipid assembly block would reduce to 0.35 in an *in planta* context, implying that LPAAT has almost half of the control in the assembly block. The experiments of Weselake *et al*. (2008) show that DGAT also has some control within the assembly, so these two enzymes together may well account for much of the control here, provided that there are no significant negative flux control coefficients in the system. The most likely source of a negative flux control coefficient would be a branch flux competing with TAG accumulation, and to generate a significant negative coefficient, it would need to have a flux that would be a significant fraction of that to TAG, since the flux ratio enters the expression for the relative values. As the TAG accumulation flux is the major carbon flux during the exponential filling phase, it seems unlikely that there could be a branch contributing significant negative control. Indeed, if there were such enzymes, their overexpression would reduce TAG yield, and their inhibition would increase it, but we know of no reports of such behaviour.

Although there were few changes in the fatty acid composition of plastid membrane lipids, acyl lipids involved in oil accumulation (DAG, PA, PC, TAG) showed significant alterations in transgenic lines (Fig. 5; Table S1). Of interest was the proportional increase in oleate at the expense of other C_18_ fatty acids in DAG, PC and TAG. By contrast, PA contained less oleate and increased amounts of the other C_18_ acids (Fig. 4). These changes may reflect increased preference for PA species containing oleate by phosphatidate phosphohydrolase, when carbon supply (and PA levels: see Fig. 5) are increased in LPAAT‐OE. We do not believe that the changes found in DAG, PC and TAG were due to the substrate preference of the nasturtium enzyme *per se*, as this enzyme uses palmitoyl‐CoA and oleoyl‐CoA equally, as judged by specificity measurements (Taylor *et al*., 2010) and the oilseed rape LPAAT was also shown to use these substrates at equivalent rates in a selectivity assay (Berneth & Frentzen, 1990).

Radiolabelling of polar lipid classes (Fig. 5) revealed small changes in their relative labelling. LPAAT‐OE showed increased labelling of PA, as might be expected when LPAAT activity was raised (Fig. 1a). PG was also better labelled perhaps because PA provided more substrate for its synthetic pathway (Gurr *et al*., 2016). These increases were at the expense of the relative labelling of PC.

Increased flow of carbon into lipid synthesis, promoted by the exogenous addition of oleate (Ramli *et al*., 2002; Tang *et al*., 2012) stimulated the relative labelling of PC from [U‐^14^C] glycerol in both azygotes and LPAAT‐OE. This might be expected since increased oil accumulation may, similarly, involve increased flux in and out of PC (Bates, 2016). By contrast, addition of diazepam caused the opposite changes to oleate addition in the azygote lines, namely a reduction in the relative labelling of PC and an increase in that of PG (Fig. 5c), whereas LPAAT‐OE lines were insensitive to diazepam addition (Figs 3, 5e), perhaps because the elevated LPAAT levels reduced the amount of inhibitor available to inhibit acyltransferases.

## Conclusions

Overexpression of LPAAT produced a significant increase in oil accumulation in embryos of *B. napus* cv DH12075. The estimated *in planta* flux control coefficient for LPAAT is *c*. 0.15, which is likely to represent a large proportion of the flux control in the lipid assembly block of enzymes of TAG synthesis. As both LPAAT and DGAT have been shown to hold significant flux control, it is likely that further increases in TAG synthesis would result from simultaneous expression of these two enzymes. This has important implications for efforts to increase oil productivity and, hence, for the agricultural industry.

## Author contributions

JLH, TF and DF conceived the experiments and interpreted data. HKW and SF performed the experiments and IAG helped with lipid analysis. AB contributed to enzyme evaluation and statistical analysis. EW, REB, E‐FM and DCT produced transgenic lines. DF, JLH and TF wrote the manuscript with contributions from all authors. HKW and SF contributed equally to this work.

## Supporting information

Please note: Wiley Blackwell are not responsible for the content or functionality of any Supporting Information supplied by the authors. Any queries (other than missing material) should be directed to the *New Phytologist* Central Office.

**Table S1** Fatty acid percentage composition of key lipid classes in 27 DAF Brassica napus line overexpressing LPAAT and an azygote control.
**Fig. S1** Detail of the T‐DNA structure (5742 bp) of pBI121/NastLPAT showing the nasturtium (*Tropaeolum majus*) *LPAAT* gene under the control of the napin promoter.
**Fig. S2** Selection of *LPAAT* transgenic lines.
**Fig. S3** Enzyme kinetic analysis.
**Notes S1** TAG kinetics and flux control estimations.
**Notes S2** Derivation of Eqn 4 (main text).
