# Supplementary material for: Increase in lysophosphatidate acyltransferase activity in oilseed rape (Brassica napus) increases seed triacylglycerol content despite its low intrinsic flux control coefficient
Source: New Phytol. 2019 Sep 14;224(2):700–11. doi: 10.1111/nph.16100 (PMC6790676; doi:10.1111/nph.16100)
Supplement: Supplementary file 1 — Table S1 Fatty acid percentage composition of key lipid classes in 27 DAF Brassica napus line overexpressing LPAAT and an azygote control. Fig. S1 Detail of the T‐DNA structure (5742 bp) of pBI121/NastLPAT showing the nasturtium (Tropaeolum majus) LPAAT gene under the control of the napin promoter. Fig. S2 Selection of LPAAT transgenic lines. Fig. S3 Enzyme kinetic analysis. Notes S1 TAG kinetics and flux control estimations. Notes S2 Derivation of Eqn 4 (main text). [file NPH-224-700-s001.pdf]

New Phytologist Supporting Information:

Increase in lysophosphatidate acyltransferase activity in oilseed rape (*Brassica napus* L.) increases seed triacylglycerol content despite its low intrinsic flux control coefficient.

Helen K. Woodfield, Stepan Fenyk, Emma Wallington, Ruth E. Bates, Alexander Brown, Irina A. Guschina, Elizabeth-France Marillia, David C. Taylor, David Fell, John L. Harwood and Tony Fawcett.

Article acceptance date: 23 July 2019.

Supporting Information Table S1: Fatty acid percentage composition of key lipid classes in 27 DAF *Brassica napus* line overexpressing LPAAT and an azygote control.

**TAG**	**2azA**			**2A**				**3azA**			**3B**		
16:0	4.9%	±	0.2%	4.6%	±	0.0%	*	5.1%	±	0.2%	5.0%	±	0.3%
18:0	3.7%	±	0.1%	3.3%	±	0.1%	***	3.8%	±	0.2%	3.6%	±	0.1%
18:1	69.1%	±	1.0%	70.9%	±	0.6%	*	66.0%	±	0.9%	67.0%	±	2.0%
18:2	13.2%	±	0.7%	12.5%	±	0.3%		14.8%	±	0.5%	14.1%	±	1.4%
18:3	5.2%	±	0.2%	5.4%	±	0.1%		6.0%	±	0.1%	5.9%	±	0.3%
20:0	1.3%	±	0.0%	0.9%	±	0.5%		1.4%	±	0.0%	1.5%	±	0.0%
20:1	1.1%	±	0.0%	1.2%	±	0.0%	*	1.3%	±	0.0%	1.3%	±	0.1%

**DAG**	**2azA**			**2A**				**3azA**			**3B**		
16:0	2.8%	±	0.6%	2.8%	±	0.2%		4.0%	±	0.4%	4.4%	±	0.4%
18:0	2.5%	±	0.3%	1.8%	±	0.1%	**	4.0%	±	0.8%	4.5%	±	2.1%
18:1	66.6%	±	3.0%	68.5%	±	0.9%		60.5%	±	1.0%	64.0%	±	3.4%
18:2	19.8%	±	1.5%	17.7%	±	0.6%	*	18.8%	±	2.7%	15.2%	±	2.2%
18:3	6.4%	±	0.3%	6.7%	±	0.4%		6.1%	±	1.1%	5.8%	±	0.6%

**MGDG**	**2azA**			**2A**				**3azA**			**3B**		
16:0	4.2%	±	3.1%	1.8%	±	0.5%		1.3%	±	0.1%	1.2%	±	0.3%
16:2	1.8%	±	0.4%	2.0%	±	0.2%		2.0%	±	0.5%	2.2%	±	0.4%
16:3	17.6%	±	4.3%	16.6%	±	2.1%		17.1%	±	4.4%	19.7%	±	3.2%
18:0	3.0%	±	1.3%	1.8%	±	0.5%		1.2%	±	0.2%	1.0%	±	0.7%
18:1	4.3%	±	2.3%	2.9%	±	0.2%		2.8%	±	0.9%	2.9%	±	0.7%
18:2	8.7%	±	1.3%	10.5%	±	0.6%	*	10.1%	±	0.7%	9.7%	±	0.4%
18:3	59.4%	±	5.4%	63.7%	±	2.0%		64.7%	±	3.0%	62.5%	±	1.8%

**PA**	**2azA**			**2A**				**3azA**			**3B**			
16:0	8.5%	±	1.0%	10.3%	±	1.4%		10.1%	±	1.8%	11.6%	±	0.6%	
18:0	3.2%	±	0.3%	4.4%	±	1.3%		3.4%	±	0.2%	4.5%	±	1.2%	
18:1	49.1%	±	2.4%	39.2%	±	3.9%	***	44.2%	±	0.4%	37.1%	±	2.2%	***
18:2	27.9%	±	1.1%	33.2%	±	0.9%	***	30.4%	±	1.1%	33.6%	±	2.9%	
18:3	10.2%	±	0.1%	11.5%	±	0.9%	*	10.0%	±	0.7%	11.5%	±	0.2%	**

**DGDG**	**2azA**			**2A**				**3azA**			**3B**			
16:0	4.8%	±	0.9%	6.1%	±	2.1%		3.8%	±	1.7%	4.7%	±	0.5%	
18:0	3.4%	±	0.4%	4.3%	±	0.7%		3.0%	±	0.6%	3.0%	±	0.7%	
18:1	4.9%	±	0.3%	5.8%	±	1.7%		5.8%	±	0.9%	5.7%	±	0.7%	
18:2	11.8%	±	0.4%	12.3%	±	0.2%		13.0%	±	0.5%	12.4%	±	0.5%	
18:3	73.4%	±	1.1%	69.7%	±	4.5%		73.2%	±	1.5%	73.1%	±	0.8%	

**PE**	**2azA**			**2A**				**3azA**			**3B**			
16:0	13.8%	±	0.7%	12.2%	±	1.4%		11.7%	±	4.4%	13.5%	±	0.8%	
18:0	8.0%	±	1.9%	4.3%	±	0.4%	*	5.0%	±	0.8%	3.2%	±	0.9%	*
18:1	28.3%	±	3.2%	30.6%	±	2.9%		24.8%	±	6.4%	27.7%	±	2.3%	
18:2	34.1%	±	3.7%	37.2%	±	2.1%		40.8%	±	3.0%	39.0%	±	2.2%	
18:3	14.2%	±	1.9%	14.5%	±	0.4%		16.7%	±	1.2%	15.5%	±	0.3%	

**PG**	**2azA**			**2A**				**3azA**			**3B**			
16:0	27.8%	±	4.0%	27.6%	±	3.4%		31.1%	±	8.0%	37.9%	±	4.0%	
18:0	9.2%	±	2.8%	5.2%	±	0.6%	*	4.6%	±	1.1%	3.9%	±	0.6%	
18:1	25.4%	±	6.1%	26.5%	±	1.7%		22.0%	±	3.1%	23.2%	±	7.5%	
18:2	24.7%	±	2.2%	27.6%	±	1.7%		28.7%	±	4.7%	23.8%	±	2.4%	
18:3	11.9%	±	1.1%	11.9%	±	0.4%		13.1%	±	2.2%	10.8%	±	1.4%	

**PC**	**2azA**			**2A**				**3azA**			**3B**			
16:0	8.3%	±	0.9%	7.0%	±	0.7%		6.9%	±	2.6%	8.0%	±	0.4%	
18:0	5.7%	±	1.0%	3.4%	±	0.5%	**	4.1%	±	0.4%	2.9%	±	0.2%	***
18:1	43.8%	±	6.0%	56.0%	±	3.7%	**	49.2%	±	4.8%	53.8%	±	4.4%	
18:2	26.3%	±	2.7%	23.2%	±	2.6%		27.1%	±	2.2%	24.4%	±	3.1%	
18:3	12.4%	±	2.0%	8.7%	±	0.8%	**	10.8%	±	0.8%	9.1%	±	0.9%	*
20:0	2.1%	±	2.3%	0.5%	±	0.1%		0.6%	±	0.0%	0.4%	±	0.0%	***

PI	2azA			2A			3azA			3B		
16:0	18.0%	±	0.7%	18.0%	±	2.1%	19.8%	±	2.3%	20.4%	±	1.8%
18:0	6.5%	±	1.1%	6.4%	±	0.9%	5.6%	±	0.5%	5.1%	±	0.6%
18:1	42.8%	±	1.6%	41.4%	±	2.4%	38.0%	±	1.6%	40.0%	±	4.2%
18:2	23.6%	±	1.8%	24.9%	±	1.8%	27.0%	±	0.9%	25.3%	±	2.5%
18:3	8.1%	±	0.5%	8.2%	±	0.4%	8.6%	±	0.2%	8.5%	±	0.5%

Mean ± sd shown (n=4). (* P<0.05, ** P<0.01, *** P<0.001 by Student's *t*-test comparing azygote and OE lines). Lipid species with an abundance of <1% are not shown. Abbreviations: TAG, triacylglycerol; DAG, diacylglycerol; MGDG, monogalactosyldiacylglycerol; PA, phosphatidic acid; DGDG, digalactosyldiacylglycerol; PE, phosphatidylethanolamine; PG, phosphatidylglycerol; PC, phosphatidylcholine; PI, phosphatidylinositol.

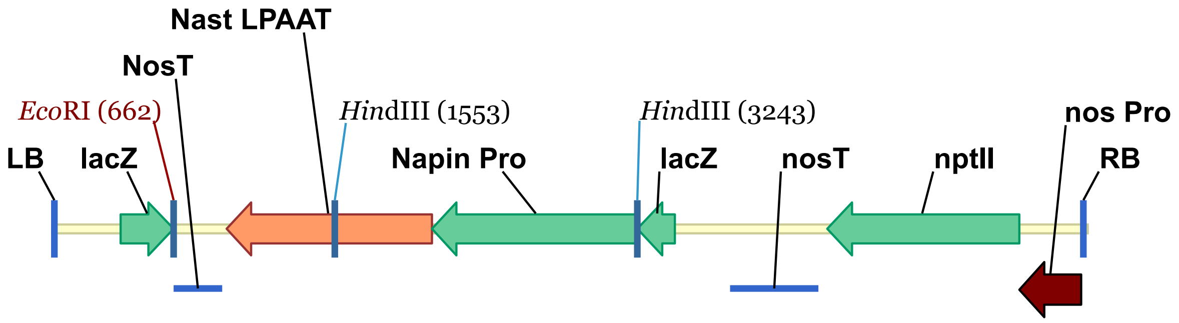

Supporting Information Figure S1: Detail of the T-DNA structure (5742 bp) of pBI121/NastLPAT showing the nasturtium (*Tropaeolum majus*) LPAAT gene under the control of the napin promoter. Information of the original cloning and characterisation of the *T. majus LPAAT* gene can be found in Taylor *et al*. (2010).

Supporting Information Figure S2: Selection of LPAAT transgenic lines

A: Amount of TAG per seed of T_1 seeds, shown as mean +/- SD of three independent biological replicates compared to azygote controls.

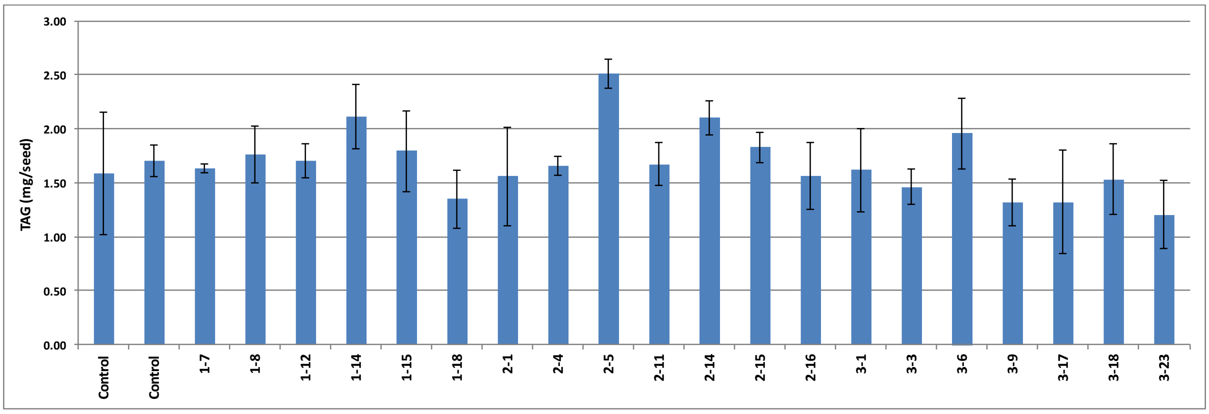

B: Analysis of the number of transgenic inserts in T_1 plants using copy number pPCR and confirmation with Southern blotting using the *nptII* gene. Hemi, hemizygous; homo, homozygous.

3-6 line

T1 plant	Copy number		Zygosity
	*npt*II	LPAAT	
3-6-122	1	1	hemi
3-6-123	1	1	hemi
3-6-128	0	1	hemi
3-6-131 (3A)	2	2	homo
3-6-137	0	0	null
3-6-137	1	1	hemi
3-6-139 (3azA)	0	0	null
3-6-141	0	0	null
3-6-142	0	0	null
3-6-143	1	1	hemi
3-6-145	1	1-2	hemi
3-6-147 (3B)	1	2	homo
3-6-148	1	1-2	hemi
3-6-151 (3azB)	0	0	null

2-14 line

T1 plant	Copy number		Zygosity
	*npt*II	LPAAT	
2-14-81	1	1	hemi
2-14-82	1-2	2	homo
2-14-85 (2A)	1	2	homo
2-14-86 (2azB)	0	0	null
2-14-89	1	1	hemi
2-14-92 (2azA)	0	0	null
2-14-94	1	1	hemi
2-14-95 (2B)	1	2	homo
2-14-99	0	0	null

Designations for plants used in the main paper are given in red.

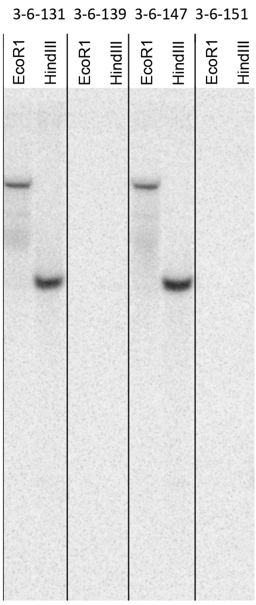

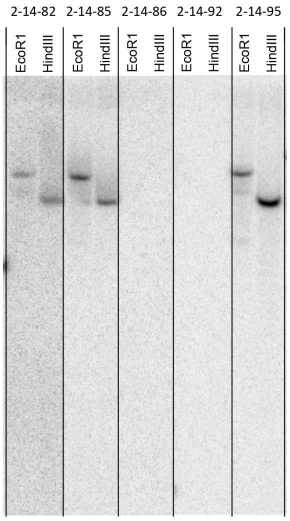

C: Sequences of primers used to verify the presence of the transgene and in copy number qPCR experiments to identify homozygous lines

**Primer name**	**Sequence 5'-3'**	**Use**
AphIII-F	GGCAATGTGCCCTTATTCC	Plasmid backbone presence PCR
AphIII-R	GCATCAGGCTCTTTCACTCC	Plasmid backbone presence PCR
LPAAT-F	TCAGCGTCTGAACGACTATCC	LPAAT presence PCR
LPAAT-R	GACCCAAACCAGATGCTGAT	LPAAT presence PCR
HMG I/Y-F	GGCAATGTGCCCTTATTCC	qPCR copy number analysis
HMG I/Y-R	GCATCAGGCTCTTTCACTCC	qPCR copy number analysis
BN10-F	AGGAACTTGCATCCTCTTTGGA	qPCR copy number analysis
BN10-R	AATAACCGGTGGAGAATGTTCAG	qPCR copy number analysis
BN10-P	VIC-ATGCTGCTGGTGCTGTGGTT-TAMRA	qPCR copy number analysis
Npt2-F	CTCCTGCCGAGAAAGTATCCA	qPCR copy number analysis
Npt2-R	GCCGGATCAAGCGTATGC	qPCR copy number analysis
Npt2-P	FAM-TGGCTGATGCAATGCGGCG-TAMRA	qPCR copy number analysis

Supporting Information Figure S3: Enzyme kinetic analysis

a) Generalized bi-substrate extension of the uni-substrate Michaelis-Menton rate equation. It was assumed that both substrates bind independently and that the binding constant for one substrate (A) is unaffected by the presence of the second substrate (B) and that release of products was not rate limiting.

$$v = \frac{V_{max} \times [A][B]}{K_{ma}[B] + K_{mb}[A] + K_{ma}K_{mb} + [A][B]}$$

b) Bi-substrate kinetics of microsomal LPAAT in regard to each substrate with 500µM of the other substrate is present in each case. Error bars show mean of three determinations +/-SEM .

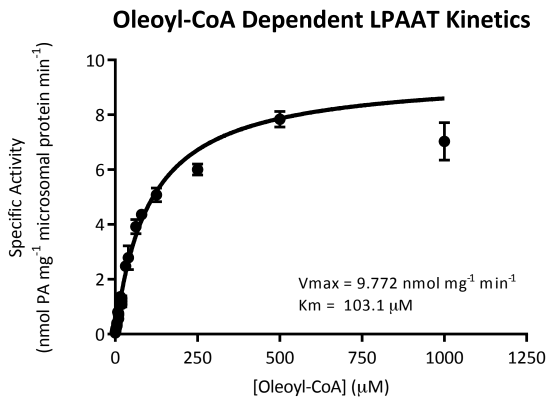

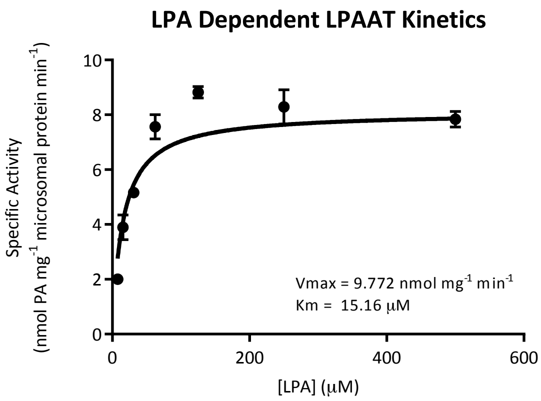

Supporting Information Note S1: TAG Kinetics and Flux Control Estimations

Kinetic equations

If the rate of increase in the amount, M, of a substance is proportional to M (i.e. first order in M), then:

$$\frac{dM}{dt} = kM$$

(Equation S1.1)

where k is the rate constant. (This rate of increase of M is also the flux J, which is therefore changing with M, but is proportional to k at any specific M.) The integrated form of this equation describes an exponential growth process:

$$M_2 = M_1 e^{k(t_2 - t_1)}$$

(Equation S1.2)

where M_1 is the amount at time t_1 and M_2 that at a subsequent time t_2. The logarithmic form of this equation:

$$\ln(M_2) = \ln(M_1) + k.\Delta t$$

(Equation S1.3)

where $\Delta t = t_2 - t_1$, shows that a log plot of M against t will be linear with slope k, as frequently used for analysis of microbial growth kinetics.

Analysis of literature data

Published results on the time course of lipid deposition in rape seeds have been noted to show an apparent exponential profile, but this does not seem to have been rigorously scrutinised. Hence graphical data in these papers was digitised using g3data (https://github.com/pn2200/g3data; version 1.5.2) and replotted as log plots (Supporting Information Note S1; Figure 1.1). Though the time scales and final seed weights differed between the studies, all showed an initial lag phase, followed by an exponential phase of variable duration that terminated relatively abruptly at close to the final seed lipid content. The mean seed lipid content at the start of the exponential phase was 0.03 ± 0.0122 mg.

A partial time course of the wild type of the plants used in this study confirmed that lipid accumulation was in the exponential phase for at least the period from 20 to 27 DAF (Supporting Information Note S1; Supporting Information Figure S1.2) with a value of the constant k within the range seen in the studies shown in Supporting Information Note S1; Supporting Information Figure S1.1.

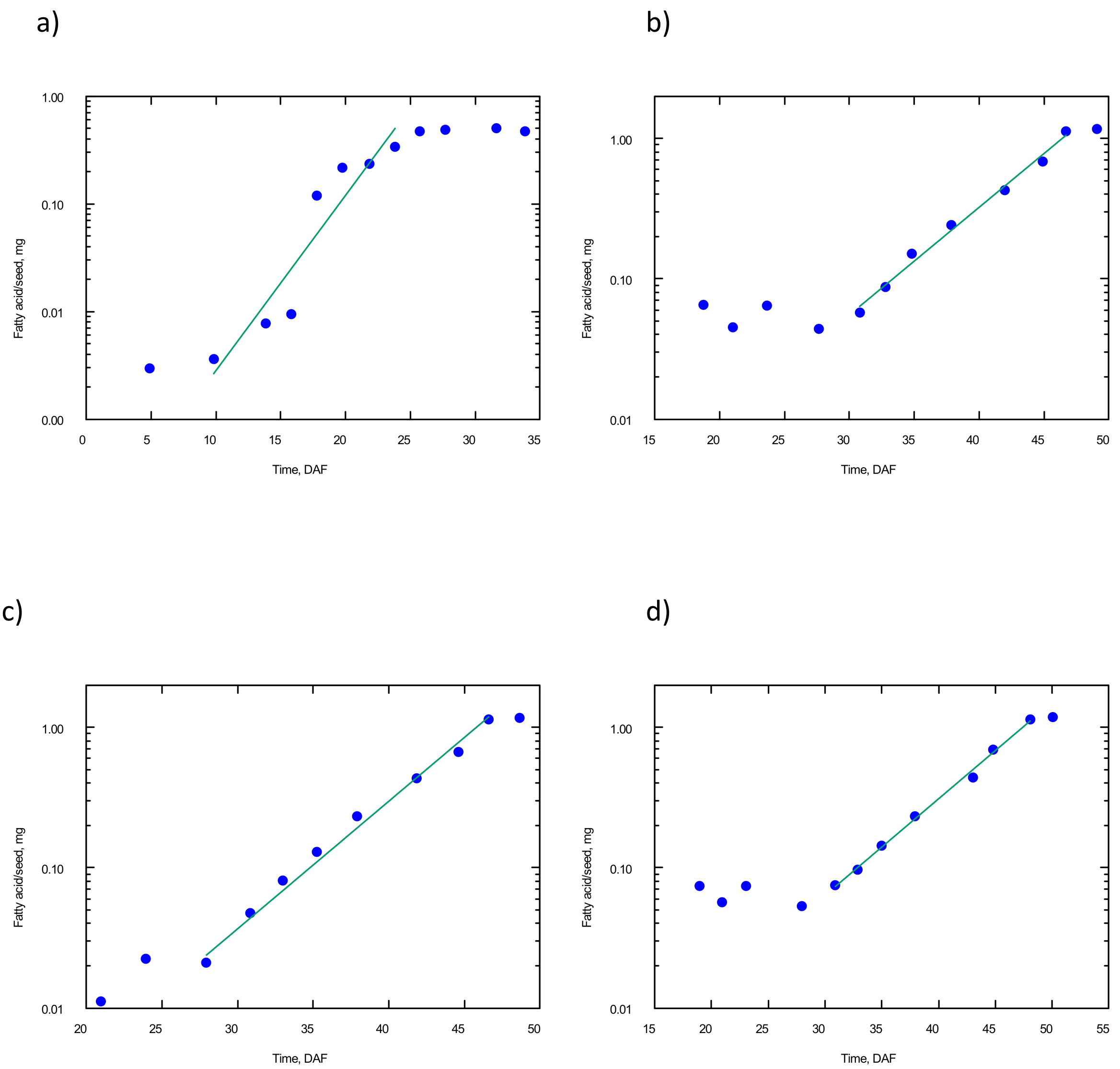

e)

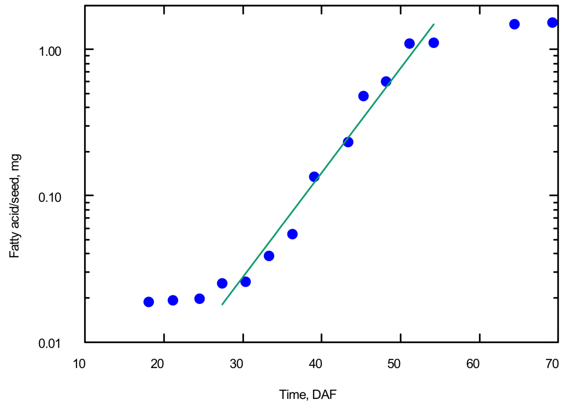

Supporting Information Note S1; Figure S1.1: Oil seed rape lipid accumulation time courses. The results are replotted in semi-log format from: a) Turnham & Northcote (1983); b) Slabas *et al.*, (1986); c) Slabas *et al.*, (1987); d) Fawcett *et al.*, (1994); and e) Hellyer *et al* (1992). The exponential phase of the time course was fitted with LibreOffice Calc.

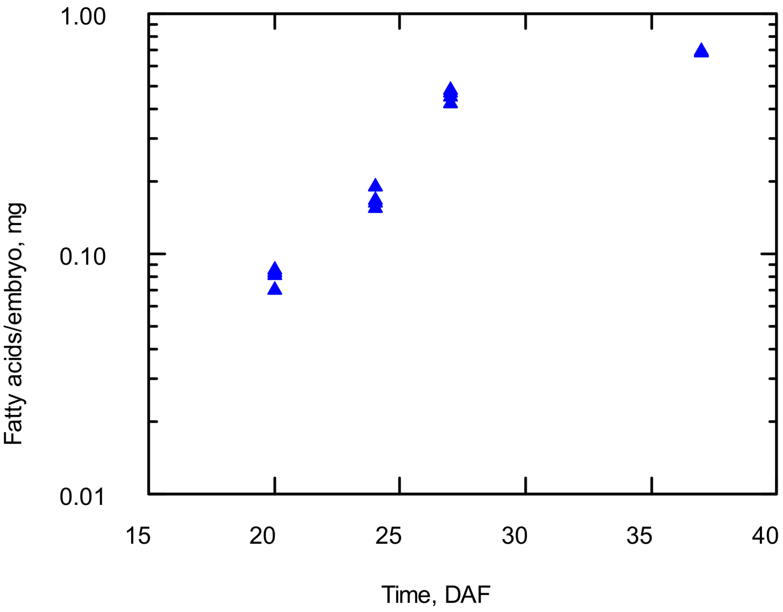

Supporting Information Note S1; Figure S1.2: Lipid deposition time course for wild-type plants used in this study. Each time point has four measurements.

Control analysis of LPAAT over-expression

Given that seed lipid accumulation is an exponential process, the rate constant k (Note 1; Equation S1.1) describes the flux to lipid, and the control coefficient for LPAAT on this constant can be shown (Fell, 2018) to be identical to the flux control coefficient of LPAAT on the lipid accumulation flux J, as defined in Eqn. (1), main text. The product $k.\Delta t$can be computed from Note 1; Equation S1.3, with M_2 as the measured final weight of TAG per seed (estimated from harvested weight, as discussed in the Main Text) and M_1 as the mean weight of lipid at the start of the exponential phase as estimated in the previous section from published data, which is assumed to be the same for both the LPAAT over-expressors and their azygote controls. On the assumption that over-expressing a Kennedy pathway enzyme has the potential to change the flux, but that there is no direct mechanism for it to affect Δt, and that a single gene insertion doubling the enzyme content is unlikely to cause other stresses on the cell, we assume that any change in $k.\Delta t$ between the azygotes and the transgenic plants reflects a change in k. Indeed, over-expressors and azygote lines were grown together and there were no visual differences in development, as judged by the timings of flowering and seed set. Hence the respective values of $k.\Delta t$ can be inserted into Eqn. (1), main text in place of J_0 and J_1 , along with the corresponding LPAAT assay values as E_1 and E_0, to give a value of the LPAAT flux control coefficient, C_E^J. Though the LPAAT assays were only taken at 27DAF, it is assumed that the ratio of the activities (the logarithm of which is what the denominator of Eqn. (1) represents) remain constant during the TAG deposition phase. The actual LPAAT activities per embryo will be increasing throughout TAG deposition as the exponential TAG accumulation occurs during exponential growth of the embryos themselves (Murphy and Cummins, 1987). In this case, enzyme activity was approximately doubled by a single gene insertion, suggesting that the activity reflects gene dosage.

However, the flux control coefficient can only be estimated accurately in this way for small changes in enzyme activity, and the increase in LPAAT activity in the transgenics made here is substantial (up to 2-fold), so instead the large change formula (Eqn. (3), Main text, Methods)

was applied. This states that the fold-change in flux, f, produced by an *r*-fold change in activity of an enzyme E with a flux control coefficient of C_E^J in the control state is:

$$f = \frac{1}{1-\frac{r-1}{r}C_E^J} \qquad \text{(Equation S1.4)}$$

This equation can be rearranged to give C_E^J in terms of the measured values of *r* (from the LPAAT assays) and f

$$C_E^J = \frac{f-1}{f}.\frac{r}{r-1}$$

(Equation S1.5),

f is obtained as the ratio of the $k.\Delta t$ values obtained from the final lipid masses of the azygotes and the over-expressers according to Eqn. S1.3, modified in the latter case to:

$$\ln(M_3) = \ln(M_1) + f.k.\Delta t$$

(Equation S1.6),

with M_3 the final weight of lipid in the seed of the over-expressers

The inevitable outcome of making a large change ΔE in the activity of E is that the control coefficient also alters. However, Equation S1.4 is indifferent whether the change in E is positive or negative, so the altered enzyme activity can be taken as the reference and the values of *r* and *f* relating it to the initial control are r'= 1/r and f' = 1/f. Substituting these in Eqn. S1.5 gives the value of the control coefficient $C_{E+\Delta E}^J$:

$$C_{E+\Delta E}^J = \frac{f-1}{r-1}$$

(Equation S1.7)

Justification for applying the large change formula to the LPAAT data

The use of the large change formula depends on the relationship between flux and enzyme activity being approximately hyperbolic. Our results give us three points on this curve: the controls with relative flux 1.0 for relative enzyme activity of 1.0, and the 2A/2B and 3A/3B

over-expressors (data in Table 1, Main text). This can be fitted by a hyperbola with parameters Q1 = 1.16 and Q2 = 0.157 (Torres et al, 1986) as shown in Fig. S1.3

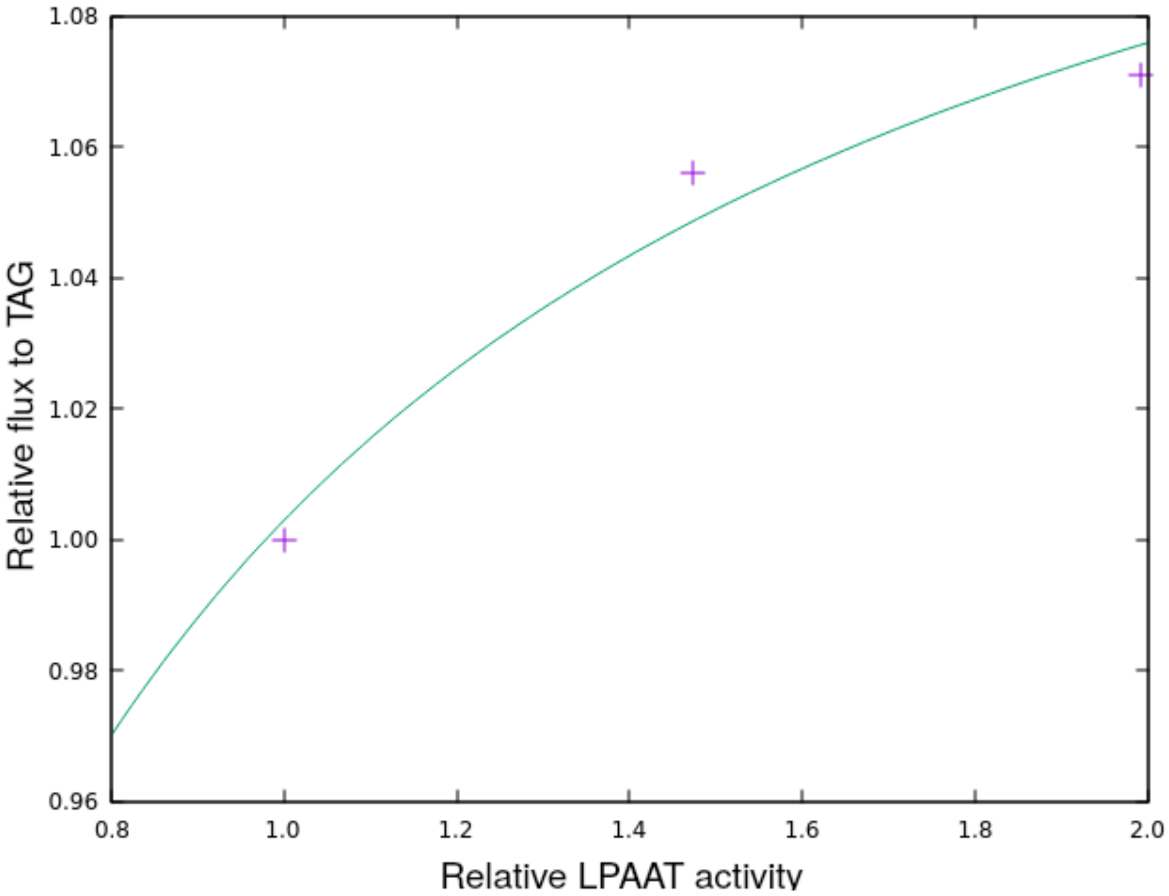

Supporting Information, Note S1. Fig S1.3. Hyperbolic relationship between LPAAT activity and flux to TAG. The plot data is derived from Table 1, Main text, and the hyperbola was fitted as described in the text above.

Dependence of flux and seed lipid accumulation on LPAAT over-expression

Given the value of the flux control coefficient in the azygote plants, it is possible to interpolate the flux response to any specific degree of overexpression of LPAAT using the large change formula (Eqn. (3), Main text, Methods and Eqn. S1.4) to calculate *f* as a function of *r*, and to use this to calculate the fold increase in lipid content L_r by:

$$L_r = e^{(f_r - 1)k\Delta t}$$

(Equation S1.8),

which is readily derived from Eqn. (S1.3)

This is plotted for comparison in (Supporting Information Note S1; Supporting Information Figure S1.4), using the value of 0.14 for the control coefficient of LPAAT and supports the claims made in the Discussion of the main paper.

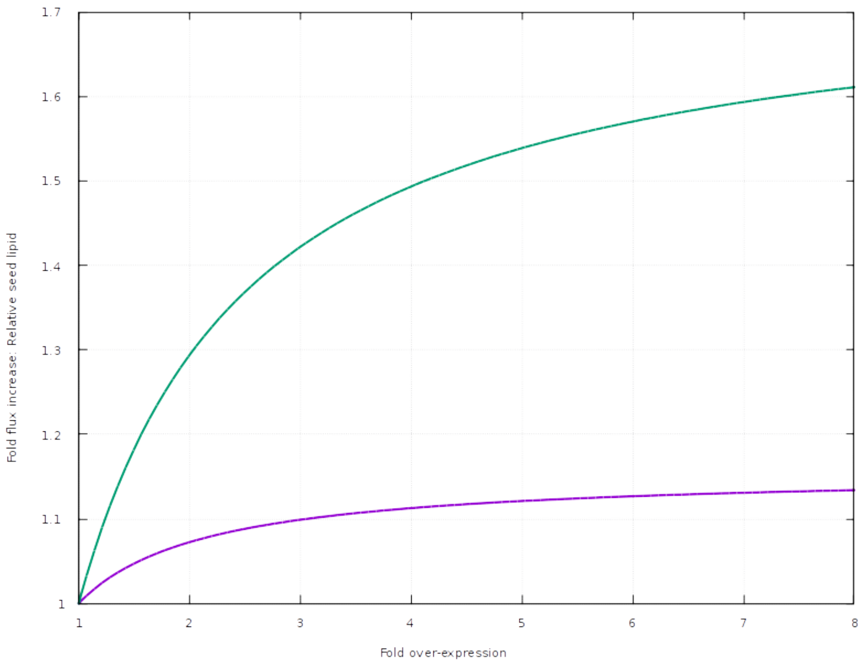

Supporting Information Note S1; Figure S1.4: Predicted effects of over-expression of LPAAT on relative flux to lipid and on final seed lipid accumulation.

The curves have been calculated using the function in Equations (S1.4) and (S1.6) for a wild-type flux control coefficient of 0.14 in order to extrapolate the curve fitted to our experiments in Fig. S1.3.

Supporting Information Note S2: Derivation of Eqn. 4 (Main text)

Eqn. (2) in the Main Text , reproduced here as Eqn. (S2.1), is the Small & Kacser (1993) function relating the expected fold change in metabolic flux, f (= J_1/J_0), as a result of an r-fold over-expression (= E_1/E_0) of an enzyme with a flux control coefficient C_E^J as:

$$f = \frac{1}{1 - \frac{r-1}{r} C_E^J}$$

(S2.1)

This can be rearranged to give the value of C_E^J in the original state as:

$$C_E^J = \frac{f-1}{f} . \frac{r}{r-1}$$

(S2.2)

Because the Eqn. (S2.1) also works for attenuation of enzyme activity, it is possible to take the over-expressed state as reference, and use Eqn. (S2.2) to calculate the flux control coefficient there. The flux ratio change would now be $f' = J_0/J_1 = 1/f$, and the enzyme activity change $r' = E_1/E_0$ = 1/r. Hence (S2.2) becomes:

$$C_{E+\Delta E}^J = \frac{f'-1}{f'} . \frac{r'}{r'-1}$$

(S2.3)

Substituting f' by $1/f$, r' by 1/r and rearranging gives Eqn. (S2.4), which is Eqn. (4) in the Main Text.

$$C_{E+\Delta E}^J = \frac{f-1}{r-1}$$

(S2.4)

References:

Fawcett T, Simon WJ, Shanklin J, Nishida I, Christie WW, Slabas AR 1994. Expression of mRNA and steady-state levels of protein isoforms of enoyl-ACP reductase from *Brassica napus. Plant Mol. Biol.* 26:155-163.

Fell DA. 2018. Metabolic Control Analysis of Exponential Growth and Product Formation. bioRxiv doi: 10.1101/485680

Murphy, DJ and Cummins, I. 1987. Biosynthesis of seed storage products during embryogenesis of rapeseed, Brassica napus. Journal of Plant Physiology 135; 6-69.

Hellyer A, Leadlay PF and Slabas AR. 1992. Induction, purification and characterisation of acyl-ACP thioesterase from developing seeds of oil seed rape (*Brassica napus*) Plant Molecular Biology 20: 763-780.

Slabas AR, Sidebottom CM, Hellyer A, Kessell RMJ, Tombs MP. 1986. Induction, purification and characterisation of NADH-specific enoyl acyl carrier protein reductase from developing seeds of oil seed rape (*Brassica napus*). *Biochimica et. Biophysica Acta* 877: 271-280

Slabas AR, Harding J, Hellyer A, Roberts P, Bambridge HE. 1987. Induction, purification and characterisation of acyl carrier protein from developing seeds of oil seed rape (*Brassica napus*). *Biochimica et. Biophysica Acta* 921: 50-59.

Small JR, Kacser H. 1993. Responses of metabolic systems to large changes in enzyme activities and effectors. 1. The linear treatment of unbranched chains. *European Journal of Biochemistry* 213, 613–624.

Taylor DC, Francis T, Lozinsky S, Hoffman T, Giblin M, Marillia E-F. 2010. Cloning and characterization of a constitutive lysophosphatidic acid acyltransferase 2 (LPAT2) gene from *Tropaeolum majus* L. *Open Plant Science Journal* 4: 7-17.

Turnham E and Northcote DH. 1983. Changes in the activity of acetyl-CoA carboxylase during rape seed formation *Biochemical Journal* 212:223-229.
